# Primary Pancreatic Head Tuberculosis: Great Masquerader of Pancreatic Adenocarcinoma

**DOI:** 10.14740/gr650w

**Published:** 2015-04-03

**Authors:** Dhaval Gupta, Jatin Patel, Chetan Rathi, Meghraj Ingle, Prabha Sawant

**Affiliations:** a Department of Gastroenterology, Lokmanya Tilak Municipal Medical College & Hospital, Sion, Mumbai, India

**Keywords:** Pancreatic tuberculosis, Pancreatic carcinoma, Endoscopic ultrasound

## Abstract

Isolated pancreatic tuberculosis (TB) is considered an extremely rare condition, even in the developing countries. Most reported cases of pancreatic TB are diagnosed after exploratory laparotomy or autopsy. Pancreatic TB is a potential mimic of invasive pancreatic malignancy and the presence of vascular invasion does not distinguish one condition from the other. Every effort should be made for the earliest diagnosis of this condition as TB is a treatable condition and it avoids unnecessary management of pancreatic carcinoma. Here we report a rare case of primary pancreatic head TB in a 58-year-old male who presented with hypodense lesion in head of pancreas with double duct sign and portal vein invasion mimicking non-resectable pancreatic carcinoma.

## Introduction

Abdominal tuberculosis (TB) is a common disease in developing countries and now it is an important differential diagnosis in developed countries also. However, isolated pancreatic TB is considered to be an extremely rare condition, even in developing countries. Most reported cases of pancreatic TB are diagnosed after exploratory laparotomy or autopsy. Every effort should be made for the earliest diagnosis of this condition as TB is a treatable condition and it avoids unnecessary management of pancreatic carcinoma. Here we report a rare case of primary pancreatic head TB mimicking pancreatic carcinoma in a 58-year-old male.

## Case Report

**Figure 1:**
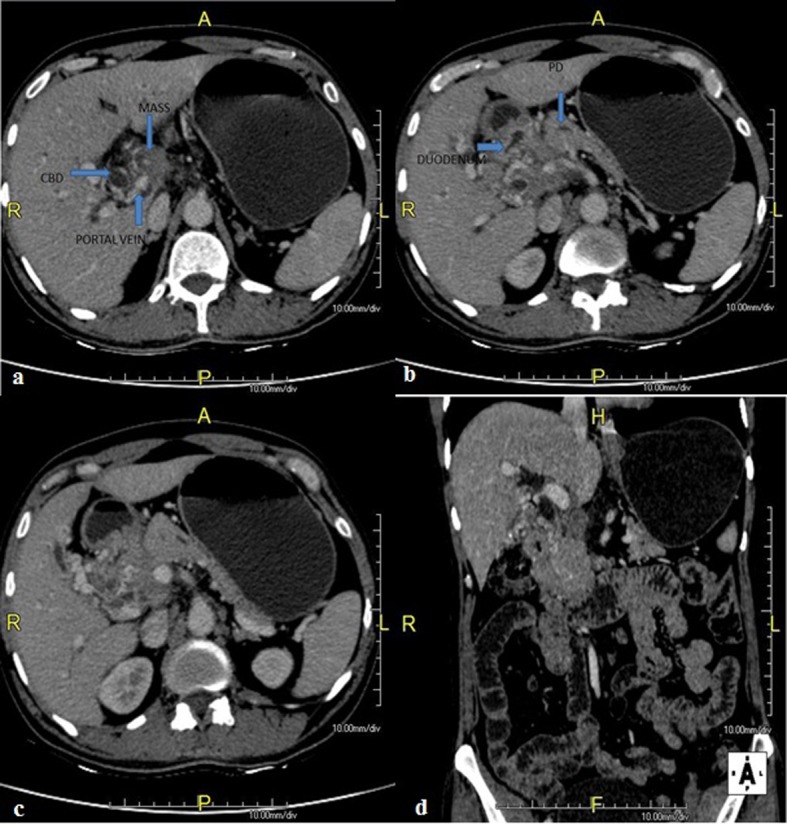
CECT scan abdomen showing hypodense lesion in pancreas head with bulky head with multiple discrete lymph nodes, largest measuring 1.3 × 1.2 cm with compression of main portal vein with presence of multiple collaterals. Pancreatic duct was dilated with 6 mm diameter in head region. Double duct sign was present.

**Figure 2:**
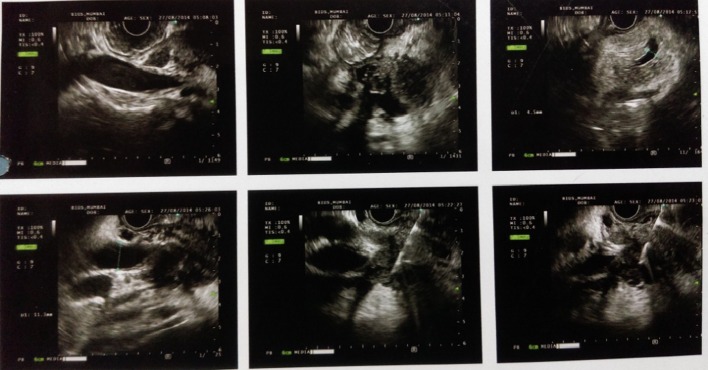
Endoscopic ultrasound (EUS) showing hypoechoic lesion in pancreas head causing dilation of CBD (11.3 mm) and PD (5.3 mm) with compression of portal vein and multiple peripancreatic and perilesional nodes present.

**Figure 3:**
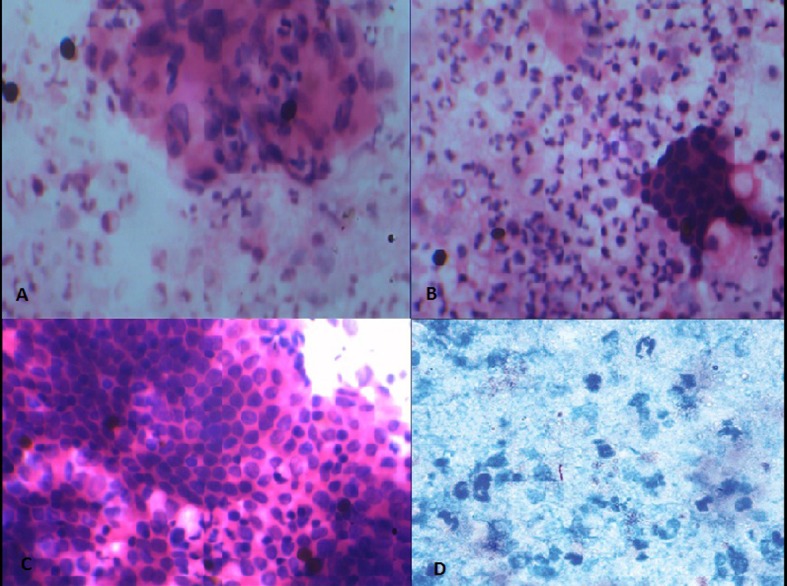
Fine needle aspiration cytology showing (A-C) epithelioid cell granulomas with Langhans giant cells with no evidence of malignancy. (D) Acid-fast bacillus stain was positive.

A 58-year-old male was admitted with pain in abdomen, weight loss and anorexia since 3 months. Abdominal pain was periumbilical, severe boring and radiating to back. Weight loss was 10 - 12 kg in last 3 months. He had occasional low grade fever and vomiting. He had no history of jaundice, altered bowel habits, gastrointestinal bleeding, and lump in abdomen. He was a chronic smoker and occasional alcoholic. On examination, there was palpable soft gall bladder. Blood investigation showed Hb 9.8 g/dL, platelet 215,000/mm^3^, leucocyte count 4,300/mm^3^, total bilirubin 1.1 mg/dL (0.2 - 1.2 mg/dL), ALT 34 IU/L (0 - 40 IU/L), AST 39 IU/L (0 - 40 IU/L) with serum alkaline phosphatase 415 (ULN 306 IU), and serum creatinine 0.9 mg/dL (0.7 - 1.2 mg/dL). We suspected pancreatic malignancy at this stage. His HIV ELISA test was negative. Chest roentgenogram was normal. Ultrasound scan of abdomen showed 3.5 × 3 × 3.9 cm hypoechoic lesion in head of pancreas with mild central and peripheral intrahepatic biliary radicles dilatation, common bile duct (CBD) diameter measured 12 mm at porta hepatis. Contrast enhanced computed tomogram (CECT) scan (Fig. 1) revealed hypodense lesion in pancreas head with bulky head. There were multiple discrete non-necrotic periportal and peripancreatic lymph nodes, largest measuring 1.3 × 1.2 cm with compression of main portal vein. There were also multiple periportal, pericholecystic collaterals present. Pancreatic duct was dilated with 6 mm diameter in head region. Double duct sign was present. These results supported our diagnosis with possibility of carcinoma of pancreas head. So we checked the serum level of CA-19.9. But it was 15.99 (ULN 37 U/mL). Endoscopic ultrasound (EUS) (Fig. 2) was done, which revealed hypoechoic lesion in pancreas head causing dilation of CBD (11.3 mm) and PD (5.3 mm). Lesion was compressing portal vein with multiple peripancreatic and perilesional nodes present. EUS guided fine needle aspiration (FNA) of pancreatic head lesion was done by using 25 gauge needle (Fig. 3). Cytology demonstrated epitheloid cell granulomas with Langhans giant cells with no evidence of malignancy. Acid-fast bacillus stain was positive. QuantiFERON^®^-TB Gold test was strongly turned out positive. Mantoux test was positive with > 20 mm diameter after 48 hours. HRCT chest was normal and did not show any evidence of old tubercular lesion. Patient’s sputum acid-fast bacillus stain and culture was negative. Patient was started anti-tubercular therapy including isoniazide (600 mg), rifampicin (450 mg), pyrazinamide (1,500 mg) and ethambutol (1,200 mg) thrice weekly schedule for 6 months according to national guideline. Patient responded very well in form of weight gain and improvement in appetite with relief from pain within 1 month of treatment. Abdominal ultrasound after 2 months showed resolution of head lesion and lymph nodes.

## Discussion

Abdominal TB generally affects bowel, particularly ileo-cecal area and lymph nodes but can involve liver or spleen [1, 2]. Pulmonary and abdominal TB coexist in 5-36% of patients [3]. Isolated pancreatic TB is rare even in high endemic countries. In India case series of 300 patients, no cases of pancreatic TB was found in surgically confirmed abdominal TB by Bhansali [1]. In another case series of 1,656 autopsies performed on TB infected patients, no case of isolated pancreatic TB was found by Auerbach [4]. Rarity of this infection is possibly explained by following observations. Pancreas is uniquely situated in retroperitoneum, protected from direct environmental exposure. Pancreatic lipases, DNAses and other enzymes appear to have antimycobacterial effects [5, 6]. Thus pancreas is relatively resistant to mycobacterial invasion, requiring a large intrapancreatic inoculum of *Mycobacterium tuberculosis* to cause pancreatic lesions [7]. Postulated routes of spread are either directly from involved peripancreatic lymph nodes or very rarely from hematogenous spread. Reported clinical manifestations include abdominal pain (75%) and anorexia with weight loss (69%), fever and night sweats (50%), whereas back pain and jaundice occur less commonly (31-40%) [8]. As the clinical and radiographic presentation mimics pancreatic cancer, preoperative diagnosis of pancreatic TB is rare. Even on EUS, pancreatic TB is not distinguishable from pancreatic malignancy and presents as hypoechoic lesions as in malignancy [9]. Even on FDG-PET, TB closely mimics pancreatic malignancy and standardized uptake values can be as high as those for malignant lesions [9, 10]. Vascular invasion of abdominal vessels is often regarded as feature of locally advanced malignancy and often been reported as point of distinction between pancreatic TB and pancreatic malignancy [11]. Our patient also had portal vein involvement with intraabdominal collaterals. Most reported cases have been diagnosed via laparoscopic biopsy or at laparotomy. There are many incidents in the past where extensive surgeries have been performed for high suspicion of periampullary carcinomas which later turned out to be TB of the pancreas [12]. EUS-FNA has emerged as an excellent tool to image and sample pancreatic lesions [13]. It is most sensitive and specific method to identify pancreatic masses and The American Joint Commission on Cancer now recommends EUS-FNA as the preferred diagnostic modality for pancreatic masses [14]. Percutaneous imaging or EUS-FNA sampling for staining, cytology, bacteriology, culture and polymerase chain reaction assay is essential for establishing the diagnosis of pancreatic TB [9, 15]. Microscopic features of TB observed on cytology are caseation necrosis, granuloma and presence of acid fast bacilli. However, it must be remembered that bacteriological confirmation may not be possible in many patients [16]. It is also widely accepted that patients with unresectable mass or patients who are poor surgical candidates should undergo FNA before deciding upon radiotherapy or chemotherapy [17]. Histological diagnosis before surgery may alter management of certain disorders like lymphoma, small cell metastasis, and TB which may not need surgery. Isolated primary pancreatic TB diagnosed by EUS-FNA is rarely reported. Centers in tuberculous endemic zone should adopt practice of doing FNA as there are no distinctive clinical, laboratory or radiological features including vascular invasion for distinguishing pancreatic TB from pancreatic cancer and a correct pre-operative histological diagnosis can avoid unnecessary surgery.

In conclusion, primary TB of pancreas is extremely rare and diagnosis is a real challenge. Pancreatic TB is a potential mimic of invasive pancreatic malignancy and the presence of vascular invasion does not distinguish one condition from the other. Appropriate investigations with multiple modalities including CT scan and EUS guided FNA or laparoscopic biopsy, diagnosis of pancreatic TB without laparotomy are possible and disease can be effectively treated with antituberculous drugs.
